# Heat-moisture-treated rice improves oral glucose tolerance by modulating serum and fecal metabolites in mice

**DOI:** 10.3389/fnut.2025.1638682

**Published:** 2025-07-28

**Authors:** Jinming Zhang, Aohua Kong, Xiaomin Chen, Mingxue Zhang, Fei Xu, Shouna Hu, Jinyu Wang, Ke Xiong

**Affiliations:** ^1^School of Public Health, Institute of Nutrition and Health, Qingdao University, Qingdao, China; ^2^College of Life Sciences, Qingdao University, Qingdao, China; ^3^Clinical Laboratory, Qingdao Cardiovascular Hospital, Qingdao, China; ^4^Qingdao Medical College, Qingdao University, Qingdao, China

**Keywords:** heat-moisture-treated rice, hyperglycemia, metabolomics, resistant starch, lysophospholipids

## Abstract

**Background/objective:**

Heat-moisture treatment (HMT) can increase the composition of resistant starch and reduce the glycemic index in rice. However, the effect of long-term HMT-rice feeding is unknown. The objective is to investigate the effect of long-term HMT-rice feeding on alleviating hyperglycemia in mice and explore potential mechanisms.

**Methods:**

In this study, HMT-rice was characterized for its X-ray diffraction pattern, in-vitro and in-vivo digestibility. In the feeding experiment, thirty C57BL/6 male mice were fed for 3 months using one of the three diets (*n* = 10 per group): a high-fat diet (HFD, containing untreated rice), an HFD supplemented using HMT-rice, or a control diet. After 3 months, the blood glucose and lipids, body weight and fat, and histopathological changes of liver and colon tissues were measured. Determination of metabolites in serum and feces was conducted by liquid chromatography tandem mass spectrometry. Association between differential serum/fecal metabolites and blood glucose/lipid parameters were determined by Spearman correlation analysis.

**Results:**

The mice in the HMT-rice group had significantly improved oral glucose tolerance and reduced serum cholesterol and body weight gain versus the HFD group. For serum metabolites, HMT-rice significantly enriched several lysophospholipids. The increase of several fecal metabolites including oxidized phospholipids and bile acid/amino acid derivatives by HFD feeding were significantly reversed by HMT-rice treatment. The changes of these serum and fecal metabolites were correlated with the changes of fasting serum glucose.

**Conclusion:**

HMT-rice significantly improved oral glucose tolerance in HFD-fed mice through the regulations of serum and fecal metabolites.

## 1 Introduction

Rice is a staple food in many countries and regions. Starch in rice is easily digested and converted into glucose by the human body, which leads to the rise of blood glucose (1, 2). A cohort study involving 132,373 participants from 21 countries reported that a high intake of rice was associated with a 20% increased risk of diabetes compared to a low intake of rice (3). Hyperglycemia induces oxidative stress and inflammation, which are detrimental to *β*-cell function and insulin sensitivity, and subsequently leads to diabetes (4–6). Physical modification of staple food to alleviate hyperglycemia is an attractive method because the process does not add chemical agents and is more appealing to the consumers (7). Also, the product may not have the side effects from taking hypoglycemic drugs (8).

HMT is an effective physical modification method to increase the composition of resistant starch (RS) in rice (9, 10). RS is a non-digestible carbohydrate and known to be beneficial for alleviating hyperglycemia through short-chain fatty acids (especially butyrate) produced by the fermentation of gut microbiota (11, 12). These short-chain fatty acids may alleviate hyperglycemia through several mechanisms (13). Increasing RS content in rice was shown to reduce adipose weight and modulate gut microbiota (14, 15).

Previous reports showed that HMT reduced the glucose response of rice after acute oral administration and had a lower glycemic index than the original rice (9). However, reducing acute glucose response does not necessarily equal long-term improvement in glycemic control and the effects of long-term HMT-rice administration on blood glucose/lipids are unknown. Also, it is not clear how HMT-rice modulates blood glucose. Metabolomics allows the detection of thousands of metabolites in biological tissues which is an efficient method for investigating biological mechanisms (16, 17) and may be helpful in revealing the modulating mechanism of HMT-rice on blood glucose.

We hypothesize that HMT-rice may alleviate blood glucose through modulating serum and fecal metabolites. We used HMT-rice to treat mice with diet-induced hyperglycemia in comparison with native rice for 3 months to demonstrate the alleviating effect of long-term HMT-rice feeding on hyperglycemia and apply metabolomic techniques to reveal the mechanism. The current work provides a foundation for applying HMT in rice processing to manage hyperglycemia.

## 2 Materials and methods

### 2.1 Preparing HMT-rice

Indica rice was obtained from Guangxi Province, China. The moisture of the rice was determined by an OHAUS MB27 moisture meter and adjusted to 25% by adding pure water. The moisture-adjusted rice was put in a sealed glass container and heated in an oven (BGZ-70, Boxun Industry & Commerce Co., Ltd.) at 110°C for 2 h. The HMT conditions were referred to previous reports (9, 18). The obtained HMT-rice and the untreated indica rice (UT-rice) were milled and used for characterization and preparation of the mice diet.

### 2.2 Chemical compositions

Lipid, ash and protein contents were measured according to the AOAC Official Method 922.06, 923.03 and 988.05, respectively (19–21). Moisture content was measured using a moisture meter (OHAUS, America). Carbohydrate content was calculated as below (22):


Carbohydrate(%)=100%−[Lipid(%)+Ash(%)+Protein(%)]

### 2.3 X-ray diffraction

X-ray diffraction (ADVANCE; Bruker, Germany) determined the crystal structures of HMT-rice and UT-rice, similar to a previous report (23). The divergent slit of CuK *α* Radiation (*λ* = 1.5405 nm) was set as 0.38 mm. The voltage and current were 40 kV and 40 mA, respectively. The diffraction angle (2θ) from 4° to 40° was scanned.

### 2.4 *In-vitro* digestion of cooked HMT- and UT-rice

The procedure was like previous reports (24, 25) with minor modifications. Forty milliliter deionized water was used to dissolve 4.5 g pancreatin (Sigma-Aldrich, United States) and the solution was heated in 37°C water with shaking for 10 min. Afterward, centrifugation at 5,000 × g for 10 min was conducted to obtain the supernatant. A total of 3.2 mL amyloglucosidase (Sigma-Aldrich, United States) was blended with 27 mL supernatant. A 0.2 g (dry weight) sample of HMT-rice or UT-rice was blended with 4 mL deionized water. The mixture was thoroughly blended and heated in boiling water for half an hour. After cooling to 37°C, 4 mL sodium acetate buffer (0.5 M, pH 5.2) and 2 mL enzyme mixture were added. The mixture was thoroughly mixed and heated in 37°C water with shaking for further reaction. At time points of 0, 20, 60, 90, and 120 min, a 0.1 mL reaction solution was blended with 90% ethanol to inactivate the enzyme. Centrifugation at 10,000 × g for 5 min was performed to obtain the supernatant. The glucose oxidase-peroxidase kit (Beijing Lideman Biochemical Co., Ltd., China) was used to measure the glucose concentration. The RS content of the samples was calculated from the glucose content at 120 min (G120) as follows:


Starch hydrolysis(%)=0.9×G120/S×100%

RS(%)=1−Starch hydrolysis(%)

where 0.9 is the conversion factor, S is the sample weight.

### 2.5 Animal experiments

#### 2.5.1 In-vivo digestibility

Ten male Sprague–Dawley rats aged 8 weeks (Beijing Vital River Co., China) were fed using a chow diet for 1 week and randomly distributed into the experiment and control group (*n* = 5 for each group). The rats were fed in an environment of 21 ± 2°C, a half-day light–dark period, and 40–60% humidity. The rats were put in standard cages (made with polycarbonate; 2 ~ 3 rats per cage) with corn cob bedding. After fasting for 12 h, the HMT-rice and UT-rice flour suspensions (7.5% w/v in water) were administered by gavage into the experiment and control group, respectively. The dose was referred to previous literature (9). Tail blood samples were obtained at 0, 30, 60, and 120 min and the blood glucose content was determined by an Omron blood glucose meter.

#### 2.5.2 HMT-rice feeding experiment

Thirty C57BL/6 male mice aged 8 weeks (Beijing Vital River Co., China) were fed using an AIN-93 M diet for 2 weeks and randomly distributed into three groups (*n* = 10 for each group): the control group, the HFD group, and the HMT-rice group. The control group used an AIN-93 M diet to feed the mice; the HFD group used a modified D12492 diet supplemented with UT-rice; and the HMT-rice group used a modified D12492 diet supplemented with HMT-rice. The supplementation ratios for UT-rice and HMT-rice were 38%. The detailed mice diet compositions were displayed in Supplementary Table A.1. The mice were fed in an environment of 21 ± 2°C, a half-day light–dark period, and 40–60% humidity for 3 months. Mice were put in standard cages (made with polycarbonate; 5 mice per cage) with corn cob bedding.

### 2.6 Blood analysis

After feeding for 11 weeks, oral glucose tolerance test (OGTT) was carried out. The mice were administered 2 g/kg body weight glucose solution by gavage after fasting overnight. At 0, 15, 30, 60, 90 and 120 min, an Omron blood glucose meter was used to record the blood glucose concentration.

The mice were euthanized via isoflurane anesthesia followed by cervical dislocation after fasting in the 12th week. Blood, fat (including epididymal, perirenal, and subcutaneous fat), liver, and intestinal tissues were collected and stored at −80°C. A 4% paraformaldehyde was used to preserve the liver and intestinal tissues. Centrifugation of the blood sample at 4,000 rpm and 4°C was able to separate the serum. An ELISA kit (Jingmei Biotech Co., Ltd., China) was used to measure blood insulin. Nanjing Jiancheng Bioengineering Institute kits were used to measure blood lipids including total cholesterol (CHO), high- and low-density lipoprotein cholesterol (HDL- and LDL-C), and total triglyceride (TG).

### 2.7 Tissue analysis

Oil red O staining was used for the liver tissues and the fat droplet area (estimating Oil red O staining score) was estimated by the Image J software (National Institutes of Health, America). Hematoxylin and eosin (H&E) staining was used for colon and liver tissues. Images were captured through an optical microscope.

### 2.8 Metabolomic analysis

Six mice in each group were randomly selected to perform the metabolomic analyses. The randomization was performed using a computer-generated random number list.

For detecting serum metabolites, serum and precooled acetonitrile were mixed (1:15 volume ratio) by vortex. Centrifugation of the mixture at 12,000 rpm and 4°C was able to separate the supernatant. After being filtered by a 0.22 μm membrane, the filtrate was injected into an Agilent 6530C liquid chromatography equipped with quadruple-time-of-flight mass spectroscopy. A Waters™ ACQUITY BEHC18 column (100 mm × 2.1 mm, 1.7 μm) was used with a gradient elution of acetonitrile and 0.1% formic acid (flow rate 0.4 mL/min). The range of 20–1,000 m/z was scanned. The flow rate and temperature of the dry gas were 7.9 L/min and 340°C, respectively. A capillary voltage of 4,500 V was used.

The fecal metabolites were measured similarly as the serum metabolites. Briefly, A 1:1 ratio of methanol and ultrapure water was precooled and used to extract 85–95 mg cecal content (5 μL extract/mg cecal content). The mixture was homogenized using zirconium beads and processed as the serum and acetonitrile mixture above.

ABf Converter (version 4.0.0) transformed the raw data to abf. MS-DIAL (version 4.38, RIKEN CSRS, University of California at Davis) was used to extract and align the peaks and reduce noise. Criteria for differentiating metabolites were P (false discovery rate-corrected) < 0.05 and fold change > 1.2 (or <0.83). The metabolites were determined by accurate mass using the Human Metabolome Database. Heatmap, metabolic function analyses, and principal component analyses graphs were generated by Metaboanalyst 5.0.

### 2.9 Statistical method

Data were shown as mean ± SD. Statistical analyses were carried out by SPSS software (Version 27.0). A one-way analysis of variance (ANOVA) test evaluated differences among the groups; and the subsequent pairwise comparison was carried out by the least square method (if the sample size was equal among the groups) or the Bonferroni method (if the sample size was not equal). For comparing blood glucose during OGTT and weight over time, a two-way repeated measured ANOVA was used. *p* < 0.05 suggested statistical significance. The association between differential metabolites and clinical indices was assessed by Spearman correlation analysis. Cytoscape (Version 3.9.0) was used to plot the interaction map between serum & fecal metabolites and blood glucose/lipid parameters.

## 3 Results

### 3.1 Characterizations of the heat-moisture-treated rice

**Figure 1:**
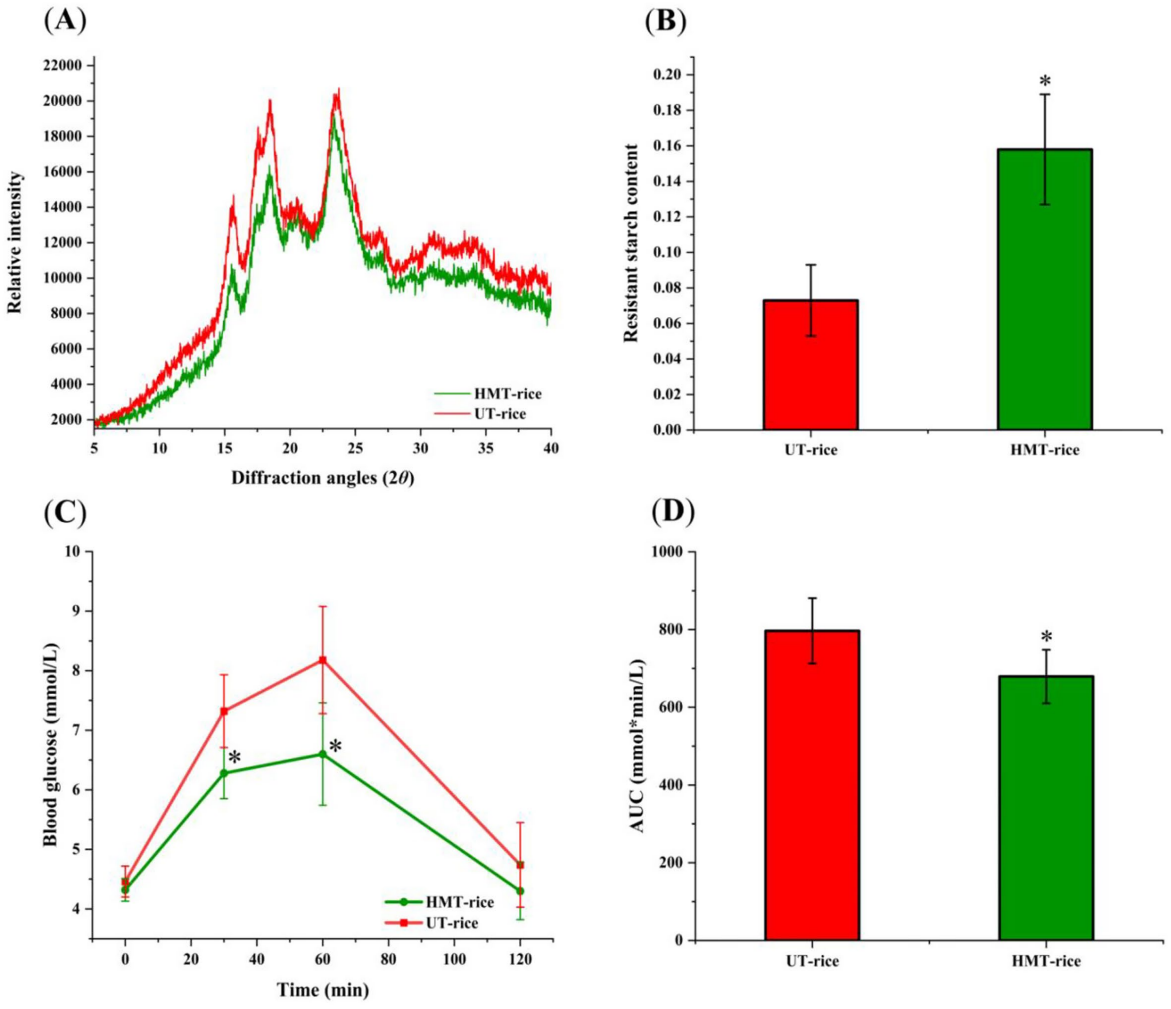
**(A)** X-ray diffraction graphs of heat-moisture-treated rice (HMT-rice) and untreated rice (UT-rice). **(B)** The composition of resistant starch in UT-rice and HMT-rice. **(C)** Mean blood glucose levels after the intake of UT-rice and HMT-rice in rats. **(D)** Area under curves (AUC) from **C**. *p < 0.05 versus UT-rice. Data were shown as mean ± standard deviation. The measurement was conducted in triplicates in **A,B**; *n* = 5 in each group for the in-vivo digestibility experiments in **C,D**.

The X-ray diffraction patterns of both UT-rice and HMT-rice (Figure 1A) displayed peaks at around 15°, 17°, 18°, 20°, and 23°, which indicated A-type crystallinity of rice starch (22, 26). The chemical composition of HMT-rice was not significantly different from that of UT-rice (Table 1).

**Table 1:** Chemical compositions of the untreated and heat-moisture treated rice.

Group	Moisture (%)	Composition (%, dry basis) of rice			
		Protein	Ash	Lipid	Carbohydrate
UT-rice	9.19 ± 1.1	7.37 ± 0.12	1.59 ± 0.68	0.93 ± 0.15	90.11 ± 0.03
HMT-rice	8.71 ± 1.1	7.30 ± 0.09	1.93 ± 0.39	1.10 ± 0.17	89.67 ± 0.14

UT-rice: untreated rice; HMT-rice: heat-moisture treated rice. All the parameters above were not significantly different between groups.

In-vitro digestion showed that the composition of resistant starch in HMT-rice was significantly higher than that in UT-rice (Figures 1B; *p* < 0.05). Consistently, in-vivo digestibility experiments (Figures 1C,D) showed that the mean blood glucose level after the intake of HMT-rice was significantly reduced compared with that after the intake of UT-rice (*p* < 0.05); and the area under curve (AUC) after the intake of HMT-rice was also significantly reduced compared with that after the intake of UT-rice (*p* < 0.05), consistent with the literature (9).

### 3.2 Effects on blood glucose and lipids

**Figure 2:**
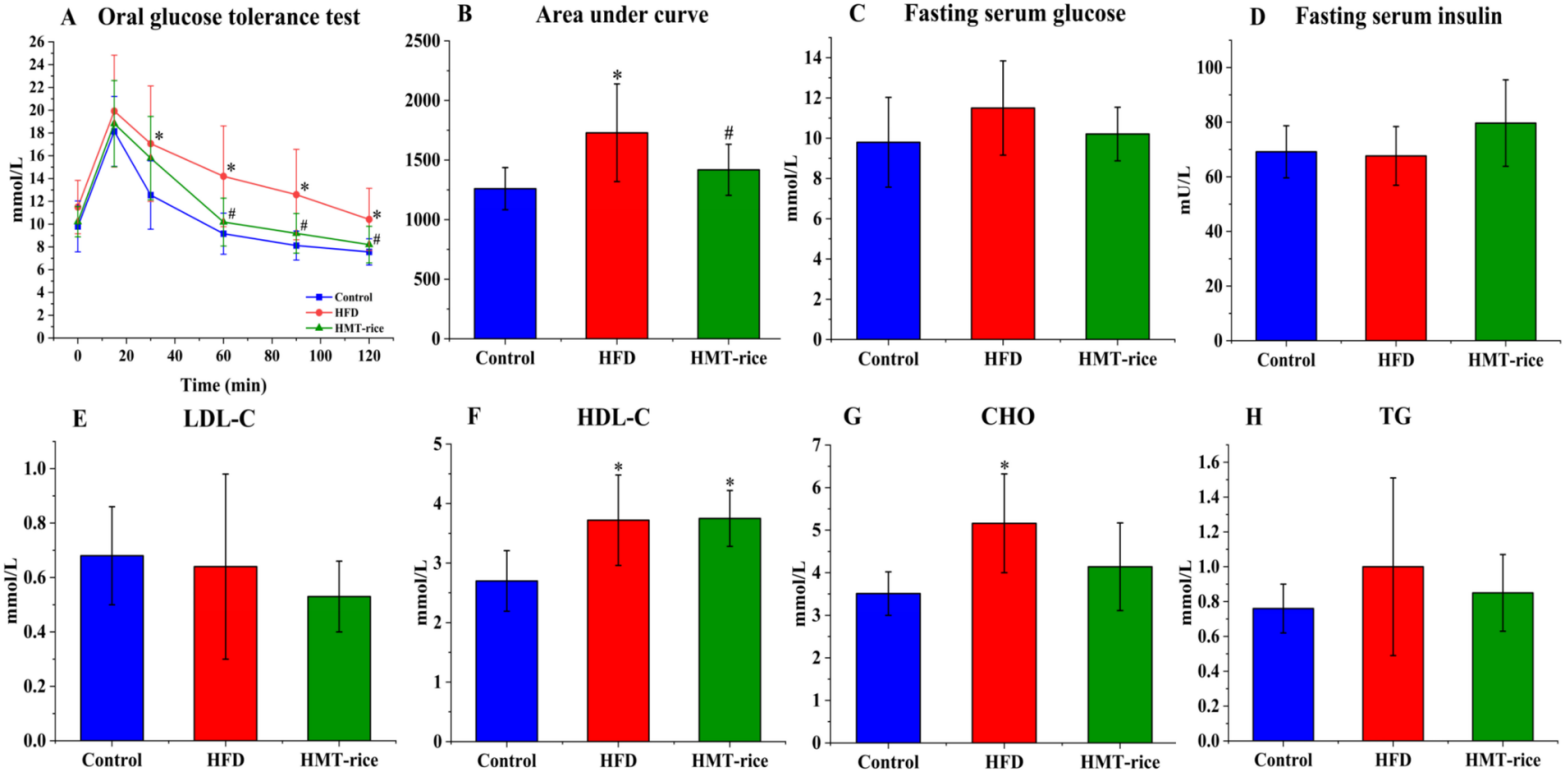
The effects of heat-moisture-treated rice (HMT-rice) on blood glucose and lipids in mice. **(A)** The oral glucose tolerance test (OGTT) curve after feeding for 11 weeks; **(B)** area under curves from OGTT; **(C)** and **(D)** fasting serum glucose and insulin; **(E)** and **(F)** low- and high-density lipoprotein cholesterol (LDL- and HDL-C); **(G)** cholesterol (CHO) **(H)** total triglycerides (TG). HFD: high-fat diet group. Data were shown as mean ± standard deviation. For **A,B**, *N* = 10 in each group; for **C–H**, *N* = 10 in the HFD group, *n* = 9 in the control and HMT-rice group. Statistical significance (*p* < 0.05) was denoted as * (versus the control group) and ^#^ (versus the HFD group).

The effects of HMT-rice on blood glucose/lipids were displayed in Figure 2. In the OGTT, the HFD group had a significantly larger AUC of the blood glucose curve than the control (*p* < 0.05); while the HMT-rice group had a significantly smaller AUC than the HFD group (*p* < 0.05). The differences in blood glucose and insulin among the three groups were not significant. The HFD group had significantly larger blood CHO and HDL-C than the control (*p* < 0.05). The three groups had similar blood TG and LDL-C.

### 3.3 Effects on obesity and histopathology

**Figure 3:**
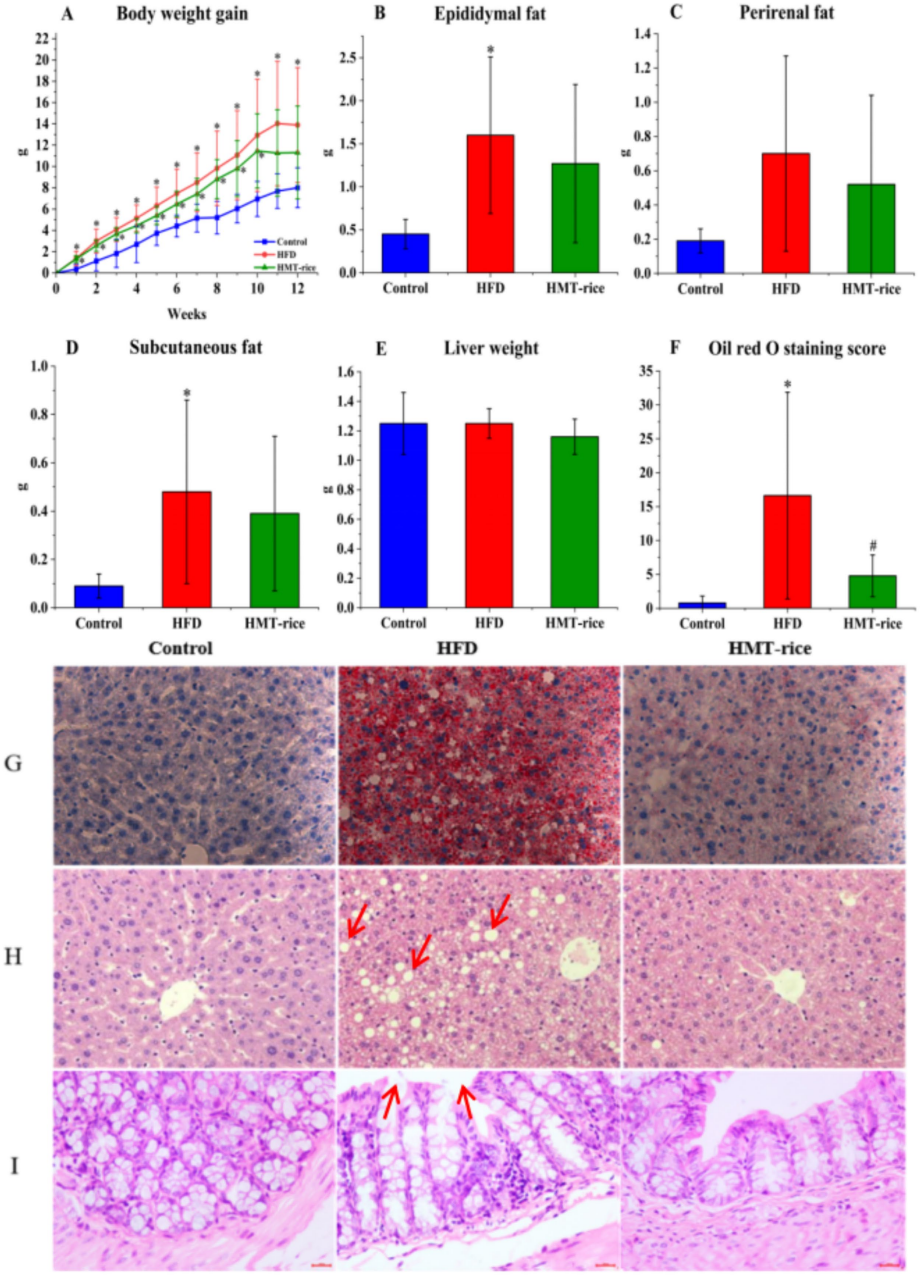
The effects of heat-moisture-treated rice (HMT-rice) on mice obesity and histology **(A)** Body weight gain; **(B–D)** epididymal, perirenal, and subcutaneous fat weight; **(E)** liver weight; **(F,G)** the Oil Red O staining and score of the liver tissues, magnified by × 400; **(H,I)** the **H**,**E** staining of the liver and colon tissues, magnified by × 400. Data were shown as mean ± standard deviation. For **A**, *N* = 10 in each group; for **B**–**F**, *N* = 10 in the control and HFD group, *n* = 9 in the HMT-rice group. Statistical significance (*p* < 0.05) was denoted as * (versus the control group) and ^#^ (versus the HFD group).

The HFD-fed mice acquired weight much more rapidly than the control mice (*p* < 0.05); the HMT-rice-treated mice acquired weight slightly slower than the HFD-fed mice without statistical significance (Figure 3A). The HFD-fed mice had significantly heavier epididymal and subcutaneous fat than the control mice (*p* < 0.05); the HMT-rice-treated mice had slightly lighter epididymal and subcutaneous fat than the HFD-fed mice without statistical significance (Figures 3B–D). The differences were not significant in perirenal fat and liver weight among the groups (Figures 3C,E).

The liver and colon histopathology were displayed in Figures 3F–I. The HFD group had a significantly higher Oil Red O staining score than the control (Figures 3F,G, *p* < 0.05); and the HMT-rice group had a significantly lower score than the HFD group (*p* < 0.05). The H&E staining indicated that more fat vacuoles appeared in the liver of the HFD-fed mice than that of the control mice; while the HMT-rice-treated mice had less fat vacuoles in the liver than the HFD-fed mice (*p* < 0.05). The H&E staining of the colon tissue suggested that the goblet cell number declined and the integrity of the epithelial structure was destroyed in the HFD-fed mice (versus the control mice). These changes were alleviated in the HMT-rice-treated mice.

### 3.4 Effects on serum and fecal metabolites

**Figure 4:**
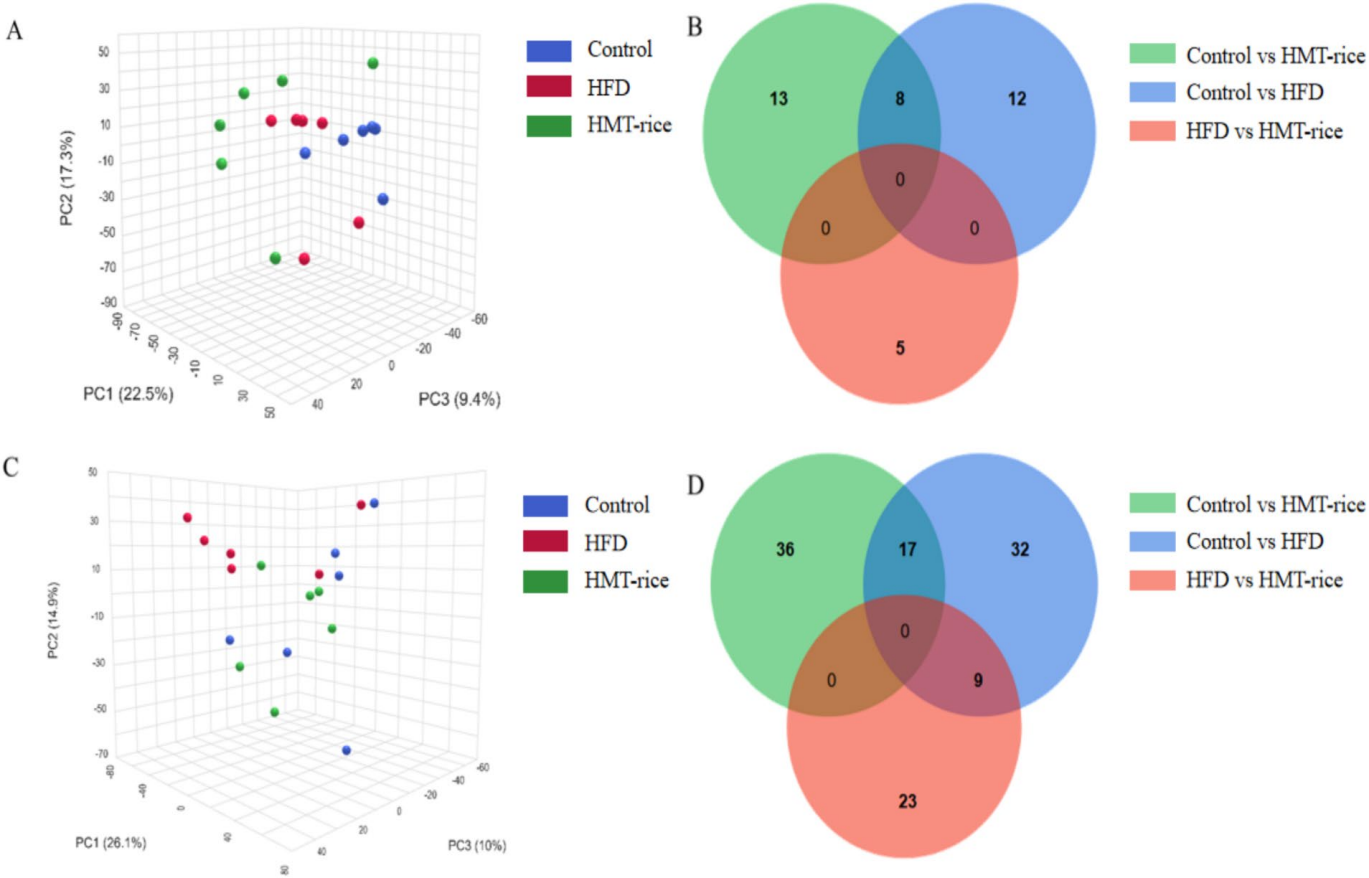
**(A)** Serum metabolic profiling shown by principal component analysis graph; **(B)** the number of distinct metabolites between groups in serum samples (shown as Venn plot); **(C)** fecal metabolic profiling shown by principal component analysis graph; **(D)** the number of distinct metabolites between groups in fecal samples (shown by Venn plot). HFD, high-fat diet; HMT-rice, heat-moisture-treated rice. *N* = 6 in each group.

Principal Component Analysis (PCA; Figure 4) plots showed clear separated metabolic profiles among the three groups (*p* < 0.05) and the detailed differential metabolites among the three groups were summarized in Supplementary Tables A.2–A.7.

**Figure 5:**
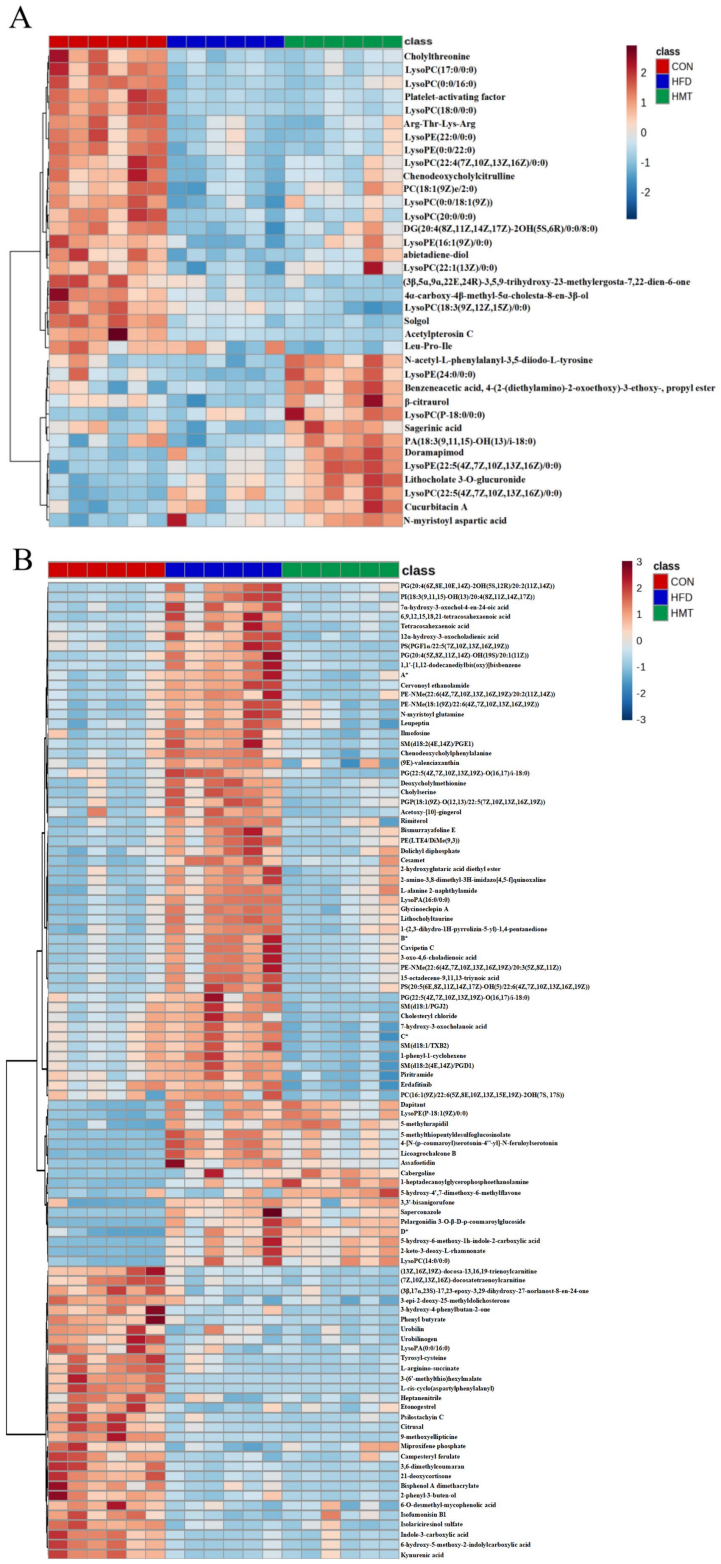
Heatmap of the differential **(A)** serum and **(B)** fecal (top 100) metabolites among the groups. *A: 2-angeloyl-9-(3-methyl-2E-pentenoyl)-2b,9a-dihydroxy-4Z,10(14)-oplopadien-3-one; *B:(2E,4E)-5-[2-methyl-2-(1,1,4,4-tetramethyl-1,2,3,4-tetrahydronaphthalene-6-yl)cyclopropyl]-3-methyl-2,4-pentadienoic acid; *C:(3R,6’Z)-3,4-dihydro-8-hydroxy-3-(6-pentadecenyl)-1H-2-benzopyran-1-one; *D: N1-(2-methoxy-4-methylbenzyl)-n2-(2-(5-methylpyridin-2-yl)ethyl)oxalamide. HFD: high-fat diet; HMT-rice: heat-moisture treated rice. N = 6 in each group.

In hierarchical cluster analysis, the serum differential metabolites were classified into two clusters according to changing trends (Figure 5A). In Cluster One, the HFD and HMT-rice group had 23 significantly decreased serum metabolites versus the control group. These metabolites mainly included lysophospholipids, bile acid, and amino acid derivatives. In Cluster Two, the HMT-rice group had 13 significantly increased serum metabolites versus the control and the HFD groups including 4 lysophospholipids, lysoPE(24:0/0:0), lysoPC(P-18:0/0:0), lysoPE(22:5(4Z,7Z,10Z,13Z,16Z)/0:0) and lysoPC(22:5(4Z,7Z,10Z,13Z,16Z)/0:0).

The top 100 fecal differential metabolites were shown in Figure 5B, which were classified into three clusters. In Cluster One, the HFD group had 53 significantly enriched fecal metabolites versus the control group which were restored in the HMT-rice group including oxidized phospholipids, long-chain fatty acids, amino acid and bile acid derivatives. In Cluster Two, the HFD and HMT-rice groups had 17 significantly increased metabolites versus the control group. In Cluster Three, the HFD and HMT-rice groups had 30 significantly decreased metabolites than the control group.

**Figure 6:**
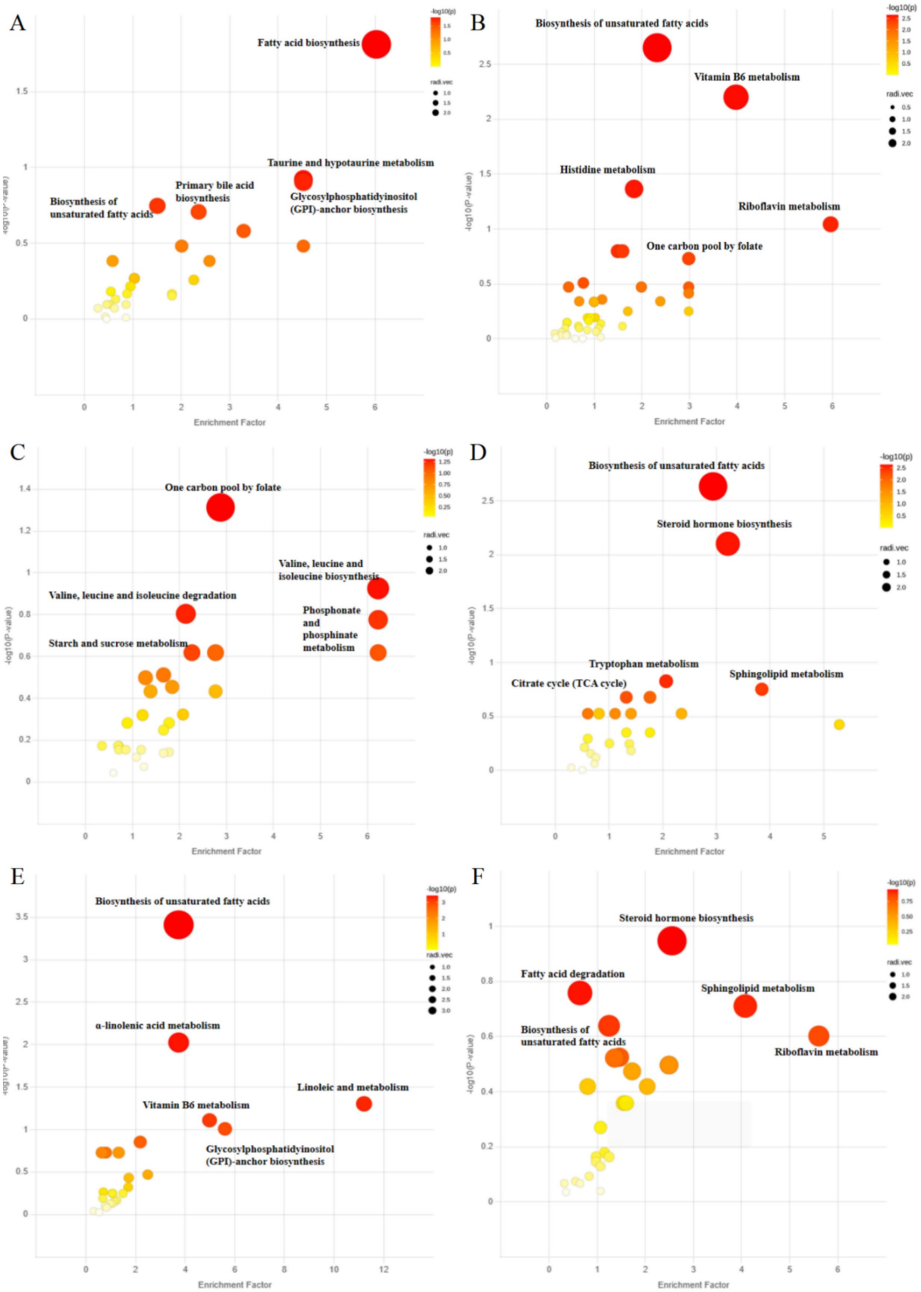
Analysis of metabolic function based on Metabolite Functional Network: **(A)** the control versus the high-fat diet (HFD) group; **(B)** the control versus the heat-moisture-treated rice (HMT-rice) groups; **(C)** the HFD versus the HMT-rice groups for the serum samples; **(D)** the control versus the HFD group, **(E)** the control versus the HMT-rice group; **(F)** the HFD versus the HMT-rice group for the fecal samples. *N* = 6 in each group.

Differential metabolic pathways between groups were determined by metabolic functional analysis based on Metabolite Functional Network (Figure 6; Supplementary Tables A.8–A.13). The differential pathways based on serum metabolites were represented in Figures 6A–C. Versus the control group, the HFD group had top distinct pathways including taurine and hypotaurine metabolism, biosynthesis of unsaturated fatty acid, and fatty acid biosynthesis. Versus the HFD group, the HMT-rice group had top distinct pathways including one carbon pool by folate, valine, leucine and isoleucine biosynthesis and degradation.

The pathways based on fecal metabolites were shown in Figures 6D–F. Versus the control group, the HFD group had top distinct pathways including biosynthesis of unsaturated fatty acids, steroid hormone biosynthesis, and sphingolipid metabolism. Versus the HFD group, the HMT-rice group had top distinct pathways including steroid hormone biosynthesis, fatty acid degradation, and sphingolipid metabolism.

### 3.5 The correlation between the clinical indicators and metabolites

**Figure 7:**
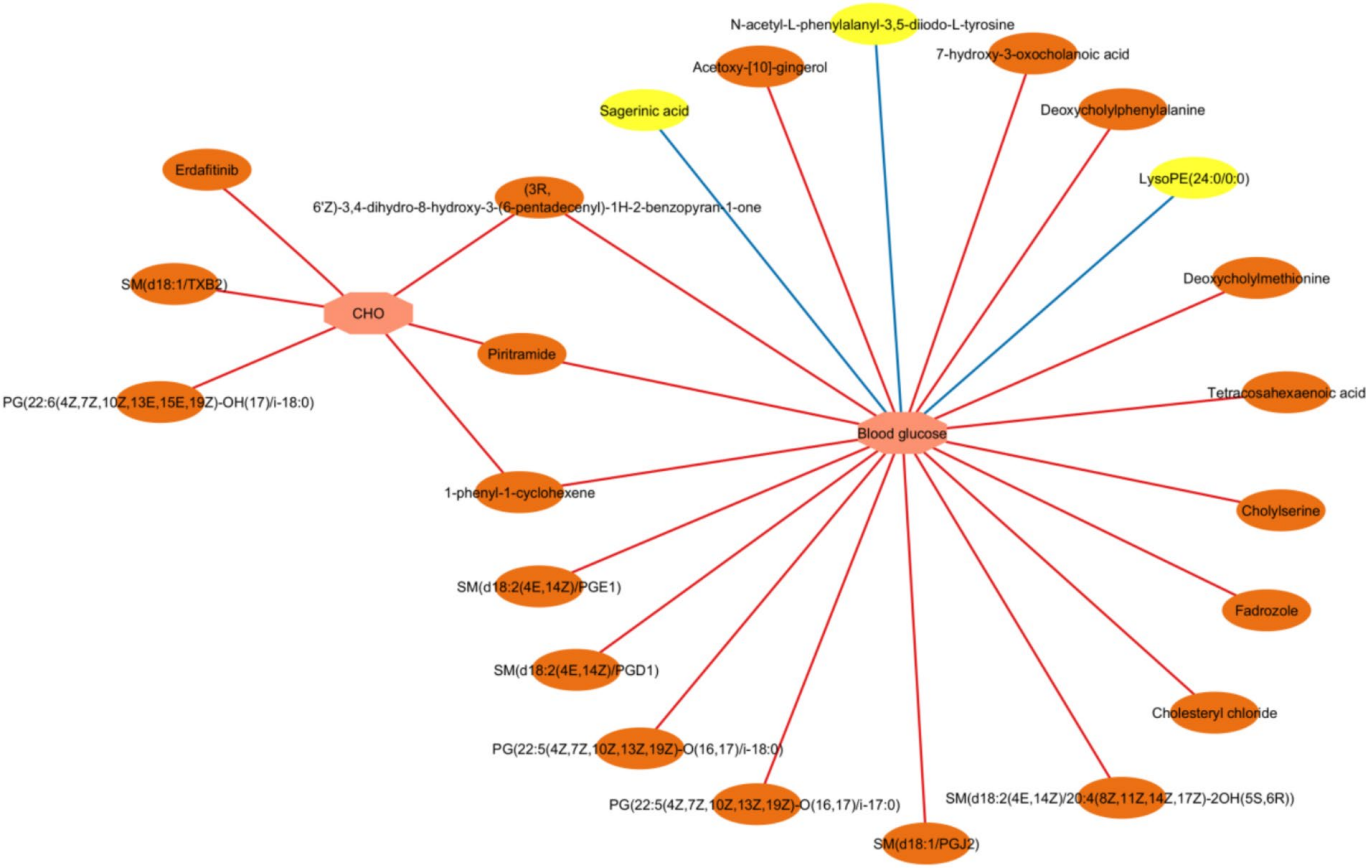
A network of interaction showing Spearman correlations between the clinical parameters and the differential fecal/serum metabolites (the heat-moisture-treated rice group vs. the high-fat diet group). CHO: cholesterol. Correlation criteria: *p* < 0.05 and correlation coefficient ≥ 0.3. Pink octagons represent blood glucose or lipid parameters; yellow ovals represent differential serum metabolites; and orange ovals represent differential fecal metabolites. Positive and negative association were shown in red and blue line, respectively. *N* = 6 in each group.

Correlation between the differential serum/fecal metabolites (HFD vs. HMT-rice) and clinical indicators was assessed by a Spearman rank correlation analysis (Figure 7; Supplementary Tables A.14, A.15). Inverse correlations were observed between fasting blood glucose and three serum metabolites including lysoPE(24:0/0:0), N-acetyl-L-phenylalanyl-3,5-diiodo-L-tyrosine, and sagerinic acid. Seventeen fecal metabolites were positively correlated with the fasting blood glucose mainly including several bile acid and amino acid derivatives, tetracosahexaenoic acid, and PG(22:5(4Z,7Z,10Z,13Z,19Z)-O(16, 17)/i-18:0). Six fecal metabolites were positively related to the CHO level.

## 4 Discussion

We are the first to study the role of serum and fecal metabolites in the effect of HMT-rice on hyperglycemia. The oral glucose tolerance of HFD-fed mice was significantly improved by HMT-rice treatment, which was associated with the regulations of serum and fecal metabolites by HMT-rice.

First, the effect of HMT-rice on hyperglycemia was associated with the regulation of serum metabolites by HMT-rice. The HMT-rice group had four significantly increased lysophospholipids versus the HFD group, including lysoPE(24:0/0:0), lysoPC(P-18:0/0:0), lysoPE(22:5(4Z,7Z,10Z,13Z,16Z)/0:0) and lysoPC(22:5(4Z,7Z,10Z,13Z,16Z)/0:0). The lysophospholipids content is critical in maintaining cell membrane structure, which promotes insulin receptor signaling and insulin-stimulated glucose uptake (27). HMT-rice treatment increased the content of lysophospholipids, which was beneficial for promoting insulin signaling and alleviating hyperglycemia.

In our metabolic functional analysis, HMT-rice significantly influenced the valine, leucine, and isoleucine biosynthesis and degradation pathways compared with the HFD group. Literature indicated strong associations between branched-chain amino acids (such as leucine, isoleucine, and valine) and insulin resistance and hyperglycemia (28–30). Leucine induces the serine phosphorylation of insulin receptor substrate 1 through stimulating mammalian target of rapamycin complex 1 and subsequently interferes insulin signaling and reduces insulin sensitivity (31). HMT-rice may also alleviate hyperglycemia through regulating the leucine, isoleucine, and valine biosynthesis and degradation pathways.

Second, the effect of HMT-rice in alleviating hyperglycemia was also associated with the regulation of HMT-rice on metabolites in feces. The HFD group had 12 significantly enriched oxidized phospholipids versus the CON group; and these oxidized phospholipids were restored to the low level in the HMT-rice group. Oxidized phospholipids were produced from the enzymatic or nonenzymatic oxidation of phospholipids including phosphatidylcholines, phosphatidylethanolamines, sphingomyelins, and phosphatidylserines, which are critical components of cell membrane and circulating lipoproteins (32, 33). Oxidized phospholipids promoted protein misfolding into amyloid fibril, which is a striking feature on the pancreas islet *β*-cell of type 2 diabetic patients compared with that of healthy controls and resulted in the death of β-cell (34, 35). In addition, oxidized phospholipids are well-known pro-inflammatory agents (32) and strong association exists between inflammation and the development of hyperglycemia (36). Consistently, the two oxidized phospholipids, [PG (22:5(4Z,7Z,10Z,13Z,19Z)-O (16, 17)/i-18:0) and SM(d18:1/TXB2)], were positively associated with the fasting serum glucose in our work. HMT-rice significantly reversed the increase of oxidized phospholipids, which was beneficial for reducing the detrimental effects of oxidized phospholipids and alleviating hyperglycemia.

In addition, HMT-rice treatment significantly reversed the increase of several bile acid and amino acid derivatives. Our spearman correlation analysis showed that four of these bile acid and amino acid derivatives were positively associated with the fasting serum glucose including chenodeoxycholylphenylalanine, deoxycholylmethionine, cholylserine, and 7-hydroxy-3-oxocholanoic acid. Consistently, previous studies showed that bile acids, aromatic amino acids, and methionine all played a role in insulin resistance and glucose homeostasis (30, 37–39). The specific roles of these bile acid and amino acid derivatives on hyperglycemia warrant further investigation.

## 5 Conclusion

In conclusion, HMT-rice treatment showed a clear effect on alleviating hyperglycemia, which may be related to its regulations of serum and fecal metabolites. Future research may investigate the detailed mechanism of HMT-rice in alleviating hyperglycemia.

## Data Availability

The original contributions presented in the study are included in the article/Supplementary material, further inquiries can be directed to the corresponding author/s.
