# Supplementary material for: Heat-moisture-treated rice improves oral glucose tolerance by modulating serum and fecal metabolites in mice
Source: Front Nutr. 2025 Jul 28;12:1638682. doi: 10.3389/fnut.2025.1638682 (PMC12336494; doi:10.3389/fnut.2025.1638682)
Supplement: Supplementary file 1 [file Table_1.docx]

Supplementary Material

**Table A.1 Compositions of the mice diets.**

|  | **Control group** | | **HFD group** | | **HMT-rice group** | |
| --- | --- | --- | --- | --- | --- | --- |
|  | **g%** | **kcal%** | **g%** | **kcal%** | **g%** | **kcal%** |
| Protein | 14.2 | 14.7 | 23.3 | 18.4 | 23.3 | 18.4 |
| Carbohydrate | 73.1 | 75.9 | 36.9 | 29.2 | 36.9 | 29.2 |
| Fat | 4.0 | 9.4 | 29.5 | 52.4 | 29.5 | 52.4 |
| Total |  | 100.0 |  | 100.0 |  | 100.0 |
| kcal/g | 3.9 |  | 5.1 |  | 5.1 |  |
|  |  |  |  |  |  |  |
| **Ingredients** | **g%** | **kcal%** | **g%** | **kcal%** | **g%** | **kcal%** |
| Casein | 14.0 | 14.5 | 18.8 | 14.9 | 18.8 | 14.9 |
| L‐cystine | 0.2 | 0.2 | 0.4 | 0.3 | 0.4 | 0.3 |
| Corn starch | 46.6 | 48.4 | 0.0 | 0.0 | 0.0 | 0.0 |
| Dextrinized corn Starch | 15.5 | 16.1 | 0.0 | 0.0 | 0.0 | 0.0 |
| Sucrose | 10.0 | 10.4 | 7.5 | 5.9 | 7.5 | 5.9 |
| UT-rice | 0.0 | 0.0 | 38 | 26.3 | 0 | 0 |
| HMT-rice | 0.0 | 0.0 | 0.0 | 0.0 | 38 | 26.3 |
| Fiber | 5.0 | 0.0 | 0.0 | 0.0 | 0.0 | 0.0 |
| Soybean oil | 4.0 | 9.4 | 2.7 | 4.8 | 2.7 | 4.8 |
| Lard | 0.0 | 0.0 | 26.4 | 47.0 | 26.4 | 47.0 |
| Mineral mix, S10026 | 0.0 | 0.0 | 1.1 | 0.0 | 1.1 | 0.0 |
| Dicalcium Phosphate | 0.0 | 0.0 | 1.4 | 0.0 | 1.4 | 0.0 |
| Calcium carbonate | 0.0 | 0.0 | 0.6 | 0.0 | 0.6 | 0.0 |
| Potassium citrate | 0.0 | 0.0 | 1.8 | 0.0 | 1.8 | 0.0 |
| Vitamin Mix, V10001 | 0.0 | 0.0 | 1.1 | 0.8 | 1.1 | 0.8 |
| Choline bitartrate | 0.3 | 0.0 | 0.2 | 0.0 | 0.2 | 0.0 |
| Mineral mix, AIN-93M-MX | 3.5 | 0.0 | 0.0 | 0.0 | 0.0 | 0.0 |
| Vitamin Mix, AIN-93-VX | 1.0 | 1.0 | 0.0 | 0.0 | 0.0 | 0.0 |
| Total | 100.0 | 100.0 | 100.0 | 100.0 | 100.0 | 100.0 |

UT-rice: untreated rice; HMT-rice: heat-moisture treated rice. Control group uses an AIN-93M diet.

**Table A.2 The differential metabolites between the control and HFD groups in the serum.**

| **Material** | **FC (Control/HFD)** | **P_adjusted_** |
| --- | --- | --- |
| abietadiene-diol | 1.66 | 0.03 |
| Solgol | 3.72 | <0.01 |
| β-citraurol | 1.27 | 0.05 |
| LysoPE(16:1(9Z)/0:0) | 1.97 | 0.02 |
| LysoPC(0:0/16:0) | 1.85 | <0.01 |
| LysoPC(17:0/0:0) | 2.06 | 0.02 |
| Cholylthreonine | 2.00 | 0.02 |
| LysoPC(0:0/18:1(9Z)) | 1.90 | <0.01 |
| LysoPC(18:0/0:0) | 3.40 | <0.01 |
| Platelet-activating factor | 3.03 | <0.01 |
| DG(20:4(8Z,11Z,14Z,17Z)-2OH(5S,6R)/0:0/8:0) | 2.92 | <0.01 |
| LysoPE(0:0/22:0) | 2.10 | 0.02 |
| LysoPE(22:0/0:0) | 2.10 | 0.03 |
| Hovenidulcigenin B | 3.68 | <0.01 |
| Chenodeoxycholylcitrulline | 2.33 | 0.02 |
| PC(18:1(9Z)e/2:0) | 2.71 | 0.03 |
| LysoPC(20:0/0:0) | 3.88 | <0.01 |
| Arg-Thr-Lys-Arg | 2.03 | 0.02 |
| LysoPC(22:4(7Z,10Z,13Z,16Z)/0:0) | 2.16 | 0.02 |
| LysoPC(22:1(13Z)/0:0) | 1.83 | <0.01 |

HFD: high-fat diet; FC: fold change.

**Table A.3 The differential metabolites between the HFD and HMT-rice groups in the serum.**

| **Material** | **FC (HMT-rice/HFD)** | **P_adjusted_** |
| --- | --- | --- |
| Benzeneacetic acid, 4-(2-(diethylamino)-2-oxoethoxy)-3-ethoxy-, propyl ester | 1.66 | 0.03 |
| LysoPE(24:0/0:0) | 2.05 | 0.03 |
| N-acetyl-L-phenylalanyl-3,5-diiodo-L-tyrosine | 1.82 | 0.03 |
| PA(18:3(9,11,15)-OH(13)/i-18:0) | 1.92 | 0.04 |
| Sagerinic acid | 2.26 | 0.04 |

HFD: high-fat diet; HMT-rice: heat-moisture treated rice; FC: fold change.

# Table A.4 The differential metabolites between the control and HMT-rice groups in the serum.

| **Material** | **FC (Control/HMT-rice)** | **P_adjusted_** |
| --- | --- | --- |
| Acetylpterosin C | 6.69 | 0.05 |
| Solgol | 5.93 | <0.01 |
| Leu-Pro-Ile | 2.39 | 0.04 |
| N-myristoyl aspartic acid | 0.57 | 0.03 |
| 9-octadecenoic acid (9Z)-, (2-hydroxy-2-oxido-1,2-oxaphospholan-4-yl)methyl ester | 3.75 | <0.01 |
| 4ɑ-carboxy-4β-methyl-5ɑ-cholesta-8-en-3β-ol | 3.48 | 0.03 |
| (3β,5ɑ,9ɑ,22E,24R)-3,5,9-trihydroxy-23-methylergosta-7,22-dien-6-one | 5.20 | 0.05 |
| LysoPC(0:0/16:0) | 1.66 | 0.03 |
| LysoPC(P-18:0/0:0) | 0.66 | 0.04 |
| LysoPC(17:0/0:0) | 1.94 | 0.03 |
| LysoPC(18:3(9Z,12Z,15Z)/0:0) | 2.66 | 0.02 |
| LysoPC(18:0/0:0) | 3.08 | <0.01 |
| Platelet-activating factor | 2.64 | <0.01 |
| LysoPE(22:5(4Z,7Z,10Z,13Z,16Z)/0:0) | 0.27 | 0.02 |
| Doramapimod | 0.30 | 0.02 |
| LysoPE(0:0/22:0) | 1.89 | 0.03 |
| LysoPE(22:0/0:0) | 2.00 | 0.04 |
| Hovenidulcigenin B | 2.49 | <0.01 |
| LysoPC(22:5(4Z,7Z,10Z,13Z,16Z)/0:0) | 0.37 | 0.03 |
| Lithocholate 3-O-glucuronide | 0.38 | 0.02 |
| Cucurbitacin A | 0.36 | 0.05 |

HMT-rice: heat-moisture treated rice; FC: fold change.

# Table A.5 The differential metabolites between the control and HFD groups in the feces.

| **Material** | **FC(Control/HFD)** | **P_adjusted_** |
| --- | --- | --- |
| 3,6-dimethylcoumaran | 4.81 | 0.03 |
| 2-keto-3-deoxy-L-rhamnonate | 0.03 | 0.04 |
| Indole-3-carboxylic acid | 5.02 | <0.01 |
| Kynurenic acid | 4.83 | <0.01 |
| 2-hydroxyglutaric acid diethyl ester | 0.26 | 0.05 |
| 1-(2,3-dihydro-1H-pyrrolizin-5-yl)-1,4-pentanedione | 0.43 | 0.03 |
| 6-hydroxy-5-methoxy-1h-indole-2-carboxylic acid | 4.95 | <0.01 |
| 2-amino-3,8-dimethyl-3H-imidazo[4,5-f]quinoxaline | 0.23 | 0.04 |
| L-alanine 2-naphthylamide | 0.39 | <0.01 |
| Rimiterol | 0.37 | <0.01 |
| Citrusal | 7.29 | 0.03 |
| L-cis-cyclo(aspartylphenylalanyl) | 7.09 | <0.01 |
| 3-(6'-methylthio)hexylmalate | 7.58 | <0.01 |
| Psilostachyin C | 4.65 | 0.04 |
| 15-octadecene-9,11,13-triynoic acid | 0.19 | 0.03 |
| L-arginino-succinate | 6.18 | 0.03 |
| Tyrosyl-cysteine | 5.67 | 0.04 |
| 6-O-desmethyl-mycophenolic acid | 3.02 | 0.04 |
| Etonogestrel | 2.90 | 0.04 |
| 9-oxo-15S-hydroxy-5Z,10Z,13E,17Z-prostatetraenoic acid | 6.14 | 0.04 |
| Licoagrochalcone B | 0.11 | <0.01 |
| 17ɑ-hydroperoxyprogesterone | 3.46 | 0.04 |
| (2E,4E)-5-[2-methyl-2-(1,1,4,4-tetramethyl-1,2,3,4-tetrahydronaphthalene-6-yl)cyclopropyl]-3-methyl-2,4-pentadienoic acid | 0.15 | 0.05 |
| 5-methylthiopentyldesulfoglucosinolate | 0.05 | <0.01 |
| N-myristoyl glutamine | 0.56 | 0.04 |
| 6,9,12,15,18,21-tetracosahexaenoic acid | 0.19 | 0.04 |
| 3-oxo-4,6-choladienoic acid | 0.19 | 0.05 |
| Cervonoyl ethanolamide | 0.40 | 0.03 |
| Cesamet | 0.23 | <0.01 |
| 7ɑ-hydroxy-3-oxochol-4-en-24-oic Acid | 0.19 | 0.04 |
| Cavipetin C | 0.15 | 0.05 |
| (9E)-valenciaxanthin | 0.59 | 0.03 |
| (7Z,10Z,13Z,16Z)-docosatetraenoylcarnitine | 2.86 | 0.04 |
| LysoPA(16:0/0:0) | 0.17 | 0.03 |
| 2-angeloyl-9-(3-methyl-2E-pentenoyl)-2b,9a-dihydroxy-4Z,10(14)-oplopadien-3-one | 0.51 | 0.05 |
| Glycinoeclepin A | 0.31 | 0.03 |
| (3β,17ɑ,23S)-17,23-epoxy-3,29-dihydroxy-27-norlanost-8-en-24-one | 2.59 | 0.04 |
| Leupeptin | 0.59 | 0.03 |
| Lithocholyltaurine | 0.23 | 0.03 |
| Cholylserine | 0.22 | 0.04 |
| Miproxifene phosphate | 2.53 | 0.05 |
| Dolichyl diphosphate | 0.40 | 0.05 |
| Deoxycholylmethionine | 0.23 | 0.04 |
| Chenodeoxycholylphenylalanine | 0.52 | 0.04 |
| 3,3'-bisanigorufone | 0.19 | 0.05 |
| Campesteryl ferulate | 3.55 | 0.03 |
| pelargonidin 3-O-β-D-p-coumaroylglucoside | 0.01 | 0.04 |
| 4-[N-(p-coumaroyl)serotonin-4''-yl]-N-feruloylserotonin | 0.01 | <0.01 |
| Isofumonisin B1 | 7.32 | 0.02 |
| Bismurrayafoline E | 0.12 | 0.04 |
| PE-NMe(18:1(9Z)/22:6(4Z,7Z,10Z,13Z,16Z,19Z)) | 0.43 | 0.03 |
| PE-NMe(22:6(4Z,7Z,10Z,13Z,16Z,19Z)/20:3(5Z,8Z,11Z)) | 0.24 | 0.05 |
| PE-NMe(22:6(4Z,7Z,10Z,13Z,16Z,19Z)/20:2(11Z,14Z)) | 0.07 | 0.04 |
| PG(20:4(6Z,8E,10E,14Z)-2OH(5S,12R)/20:2(11Z,14Z)) | 0.07 | 0.04 |
| PS(20:5(6E,8Z,11Z,14Z,17Z)-OH(5)/22:6(4Z,7Z,10Z,13Z,16Z,19Z)) | 0.09 | 0.04 |
| PI(18:3(9,11,15)-OH(13)/20:4(8Z,11Z,14Z,17Z)) | 0.08 | 0.04 |
| PE(LTE4/DiMe(9,3)) | 0.14 | 0.04 |
| PGP(18:1(9Z)-O(12,13)/22:5(7Z,10Z,13Z,16Z,19Z)) | 0.30 | 0.04 |

HFD: high-fat diet; FC: fold change.

# Table A.6 The differential metabolites between the HFD and

**HMT-rice groups in the feces.**

| **Material** | **FC(HMT-rice/HFD)** | **P_adjusted_** |
| --- | --- | --- |
| 1-phenyl-1-cyclohexene | 0.40 | <0.01 |
| Fadrozole | 0.43 | 0.04 |
| 15-octadecene-9,11,13-triynoic acid | 0.19 | 0.03 |
| 1,1'-[1,12-dodecanediylbis(oxy)]bisbenzene | 0.31 | 0.02 |
| Tetracosahexaenoic acid | 0.16 | 0.03 |
| (3R, 6'Z)-3,4-dihydro-8-hydroxy-3-(6-pentadecenyl)-1H-2-benzopyran-1-one | 0.43 | <0.01 |
| Cervonoyl ethanolamide | 0.33 | <0.01 |
| 12a-hydroxy-3-oxocholadienic acid | 0.26 | 0.02 |
| 7-hydroxy-3-oxocholanoic acid | 0.43 | <0.01 |
| Acetoxy-[10]-gingerol | 0.19 | <0.01 |
| Piritramide | 0.32 | <0.01 |
| 2-(2-methylbutanoyl)-9-(3-methyl-2E-pentenoyl)-2b,9a-dihydroxy-4Z,10(14)-oplopadien-3-one | 0.45 | 0.03 |
| Erdafitinib | 0.38 | <0.01 |
| Cholesteryl chloride | 0.36 | 0.02 |
| Cholylserine | 0.21 | 0.02 |
| Deoxycholylmethionine | 0.22 | <0.01 |
| Ilmofosine | 0.44 | <0.01 |
| Deoxycholylphenylalanine | 0.30 | 0.03 |
| Chenodeoxycholylphenylalanine | 0.43 | <0.01 |
| SM(d18:2(4E,14Z)/20:4(8Z,11Z,14Z,17Z)-2OH(5S,6R)) | 0.24 | <0.01 |
| SM(d18:1/PGJ2) | 0.11 | <0.01 |
| SM(d18:2(4E,14Z)/PGD1) | 0.15 | 0.03 |
| SM(d18:2(4E,14Z)/PGE1) | 0.21 | 0.02 |
| SM(d18:1/TXB2) | 0.18 | <0.01 |
| PG(22:5(4Z,7Z,10Z,13Z,19Z)-O(16,17)/i-17:0) | 0.11 | 0.02 |
| PE-NMe(22:6(4Z,7Z,10Z,13Z,16Z,19Z)/20:3(5Z,8Z,11Z)) | 0.15 | 0.03 |
| PC(16:1(9Z)/22:6(5Z,8E,10Z,13Z,15E,19Z)-2OH(7S, 17S)) | 0.32 | 0.04 |
| PG(22:5(4Z,7Z,10Z,13Z,19Z)-O(16,17)/i-18:0) | 0.07 | 0.04 |
| PG(22:6(4Z,7Z,10Z,13E,15E,19Z)-OH(17)/i-18:0) | 0.38 | <0.01 |
| PG(20:4(5Z,8Z,11Z,14Z)-OH(19S)/20:1(11Z)) | 0.06 | 0.03 |
| PS(PGF1ɑ/22:5(7Z,10Z,13Z,16Z,19Z)) | 0.10 | 0.02 |
| PGP(18:1(9Z)-O(12,13)/22:5(7Z,10Z,13Z,16Z,19Z)) | 0.25 | 0.02 |

HFD: high fat diet; HMT-rice: heat-moisture treated rice; FC: fold change.

**Table A.7 The differential metabolites between the control and HMT-rice groups in the feces.**

| **Material** | **FC(Control/HMT-rice)** | **P_adjusted_** |
| --- | --- | --- |
| Heptanenitrile | 2.20 | 0.04 |
| 3,6-dimethylcoumaran | 7.35 | 0.02 |
| 2-phenyl-3-buten-ol | 4.96 | 0.03 |
| 2-keto-3-deoxy-L-rhamnonate | 0.04 | <0.01 |
| Indole-3-carboxylic acid | 4.37 | 0.02 |
| Phenyl butyrate | 5.54 | 0.03 |
| 3-hydroxy-4-phenylbutan-2-one | 6.66 | 0.04 |
| Kynurenic acid | 4.00 | <0.01 |
| 5-hydroxy-6-methoxy-1h-indole-2-carboxylic acid | 0.03 | <0.01 |
| 6-hydroxy-5-methoxy-1h-indole-2-carboxylic acid | 4.21 | <0.01 |
| indole-3-acetyl-alanine | 7.11 | 0.05 |
| Citrusal | 14.17 | <0.01 |
| L-cis-cyclo(aspartylphenylalanyl) | 7.09 | <0.01 |
| 3-(6'-methylthio)hexylmalate | 7.58 | <0.01 |
| Taraxinic acid | 6.78 | 0.05 |
| Psilostachyin C | 4.99 | 0.04 |
| 9-methoxyellipticine | 9.81 | <0.01 |
| Acetylpterosin C | 3.66 | 0.04 |
| L-arginino-succinate | 22.24 | <0.01 |
| Tyrosyl-cysteine | 14.47 | <0.01 |
| 5-hydroxy-4',7-dimethoxy-6-methylflavone | 0.23 | 0.04 |
| Panaquinquecol 6 | 6.14 | 0.04 |
| 9-oxo-15S-hydroxy-5Z,10Z,13E,17Z-prostatetraenoic acid | 12.51 | 0.03 |
| N1-(2-methoxy-4-methylbenzyl)-n2-(2-(5-methylpyridin-2-yl)ethyl)oxalamide | 0.16 | <0.01 |
| 21-deoxycortisone | 6.33 | <0.01 |
| 2-hydroxymethyl-17ɑ-methylandrosta-1,4-diene-11ɑ,17β-diol-3-one | 5.84 | 0.02 |
| Bisphenol A dimethacrylate | 12.08 | 0.04 |
| 4-ethylnaphthalen-1-yl-(1-pentylindol-3-yl)methanone | 4.90 | <0.01 |
| Assafoetidin | 0.06 | <0.01 |
| 17-phenyl-18,19,20-trinor-prostaglandin D2 | 5.73 | <0.01 |
| Tryptophyl-tryptophan | 0.16 | <0.01 |
| 5-methylurapidil | 0.20 | 0.04 |
| LysoPA(0:0/16:0) | 4.30 | 0.03 |
| (7Z,10Z,13Z,16Z)-docosatetraenoylcarnitine | 5.70 | <0.01 |
| (13Z,16Z,19Z)-docosa-13,16,19-trienoylcarnitine | 6.35 | 0.02 |
| 7-chloro-3,3',4',5,6,8-hexamethoxyflavone | 9.92 | 0.04 |
| Isolariciresinol sulfate | 4.56 | 0.02 |
| Cabergoline | 0.05 | <0.01 |
| (3β,5ɑ,9ɑ,22E,24R)-3,5,9-trihydroxy-23-methylergosta-7,22-dien-6-one | 5.79 | 0.03 |
| (3β,17ɑ,23S)-17,23-epoxy-3,29-dihydroxy-27-norlanost-8-en-24-one | 2.63 | 0.02 |
| 3-epi-2-deoxy-25-methyldolichosterone | 3.29 | <0.01 |
| LysoPE(P-18:1(9Z)/0:0) | 0.16 | 0.03 |
| LysoPC(14:0/0:0) | 0.12 | 0.03 |
| 1-heptadecanoylglycerophosphoethanolamine | 0.07 | <0.01 |
| LysoPE(22:6(4Z,7Z,10Z,13Z,16Z,19Z)/0:0) | 5.98 | 0.03 |
| LysoPC(20:5(5Z,8Z,11Z,14Z,17Z)/0:0) | 0.01 | <0.01 |
| Dapitant | 0.15 | 0.02 |
| LysoPC(22:6(4Z,7Z,10Z,13Z,16Z,19Z)/0:0) | 3.75 | 0.05 |
| pelargonidin 3-O-β-D-p-coumaroylglucoside | 0.01 | <0.01 |
| Urobilinogen | 3.83 | 0.05 |
| Urobilin | 3.19 | 0.03 |
| 4-[N-(p-coumaroyl)serotonin-4''-yl]-N-feruloylserotonin | 0.01 | 0.04 |
| Saperconazole | 0.04 | 0.04 |

HMT-rice: heat-moisture treated rice; FC: fold change.

# Table A.8 The differential metabolic pathway between the HFD and control group in the serum.

| **Pathway** | **Total** | **Hits (sig.)** | **Expected** | **P** | **Enrichment factor** |
| --- | --- | --- | --- | --- | --- |
| Fatty acid biosynthesis | 9 | 3 | 0.50 | 0.015 | 6.03 |
| Primary bile acid biosynthesis | 46 | 6 | 2.54 | 0.20 | 2.36 |
| Biosynthesis of unsaturated fatty acids | 36 | 3 | 1.99 | 0.18 | 1.51 |
| Taurine and hypotaurine metabolism | 8 | 2 | 0.44 | 0.12 | 4.52 |
| Glycosylphosphatidylinositol (GPI)-anchor biosynthesis | 4 | 1 | 0.22 | 0.12 | 4.52 |

HFD: high-fat diet.

| **Pathway** | **Total** | **Hits** | **Expected** | **P** | **Enrichment factor** |
| --- | --- | --- | --- | --- | --- |
| One carbon pool by folate | 26 | 3 | 1.04 | 0.049 | 2.87 |
| Valine, leucine and isoleucine degradation | 35 | 3 | 1.41 | 0.16 | 2.14 |
| Valine, leucine and isoleucine biosynthesis | 8 | 2 | 0.32 | 0.12 | 6.23 |
| Phosphonate and phosphinate metabolism | 4 | 1 | 0.16 | 0.17 | 6.23 |
| Starch and sucrose metabolism | 11 | 1 | 0.44 | 0.24 | 2.26 |

# Table A.9 The differential metabolic pathway between the HMT-rice and HFD group in the serum.

HMT-rice: heat-moisture treated rice; HFD: high-fat diet.

# Table A.10 The differential metabolic pathway between the HMT-rice and control group in the serum.

| **Pathway** | **Total** | **Hits** | **Expected** | **P** | **Enrichment factor** |
| --- | --- | --- | --- | --- | --- |
| Biosynthesis of unsaturated fatty acids | 36 | 7 | 3.0187 | 0.002 | 2.32 |
| Vitamin B6 metabolism | 9 | 3 | 0.75 | 0.006 | 3.98 |
| One carbon pool by folate | 26 | 4 | 2.18 | 0.043 | 1.83 |
| Riboflavin metabolism | 4 | 2 | 0.34 | 0.09 | 5.96 |
| Histidine metabolism | 16 | 2 | 1.34 | 0.16 | 1.49 |

HMT-rice: heat-moisture treated rice.

# Table A.11 The differential metabolic pathway between the HFD and control group in the feces

| **Pathway** | **Total** | **Hits** | **Expected** | **P** | **Enrichment factor** |
| --- | --- | --- | --- | --- | --- |
| Biosynthesis of unsaturated fatty acids | 36 | 5 | 1.70 | 0.002 | 2.64 |
| Steroid hormone biosynthesis | 79 | 12 | 3.74 | 0.008 | 3.21 |
| Tryptophan metabolism | 41 | 4 | 1.94 | 0.15 | 2.06 |
| Sphingolipid metabolism | 11 | 2 | 0.52 | 0.18 | 3.85 |
| Citrate cycle (TCA cycle) | 16 | 1 | 0.76 | 0.21 | 1.32 |

HFD: high-fat diet.

# Table A.12 The differential metabolic pathway between the HMT-rice and HFD group in the feces.

| **Pathway** | **Total** | **Hits** | **Expected** | **P** | **Enrichment factor** |
| --- | --- | --- | --- | --- | --- |
| Steroid hormone biosynthesis | 79 | 9 | 3.52 | 0.11 | 2.55 |
| Sphingolipid metabolism | 11 | 2 | 0.49 | 0.19 | 4.08 |
| Biosynthesis of unsaturated fatty acids | 36 | 2 | 1.61 | 0.23 | 1.25 |
| Fatty acid degradation | 35 | 1 | 1.56 | 0.17 | 0.64 |
| Riboflavin metabolism | 4 | 1 | 0.18 | 0.25 | 5.61 |
| Steroid hormone biosynthesis | 79 | 9 | 3.52 | 0.11 | 2.55 |

HMT-rice: heat-moisture treated rice; HFD: high-fat diet.

# Table A.13 The differential metabolic pathway between the HMT-rice and control group in the feces.

| **Pathway** | **Total** | **Hits** | **Expected** | **P** | **Enrichment factor** |
| --- | --- | --- | --- | --- | --- |
| Biosynthesis of unsaturated fatty acids | 36 | 6 | 1.61 | 0.0004 | 3.74 |
| ɑ-linolenic acid metabolism | 12 | 2 | 0.54 | 0.009 | 3.74 |
| Linoleic acid metabolism | 4 | 2 | 0.18 | 0.05 | 11.21 |
| Vitamin B6 metabolism | 9 | 2 | 0.4 | 0.08 | 4.98 |
| Glycosylphosphatidylinositol (GPI)-anchor biosynthesis | 4 | 1 | 0.18 | 0.1 | 5.61 |

HMT-rice: heat-moisture treated rice.

# Table A.14 Spearman rank correlation results between the blood glucose/lipid parameters and the differential fecal metabolites.

| **Blood glucose/lipid parameter** | **Differential fecal metabolites** | **P** | **r** |
| --- | --- | --- | --- |
| Area under curve | 1-phenyl-1-cyclohexene | 0.53 | 0.20 |
| Area under curve | Fadrozole | 0.97 | 0.01 |
| Area under curve | 15-octadecene-9,11,13-triynoic acid | 0.70 | 0.13 |
| Area under curve | 1,1'-[1,12-dodecanediylbis(oxy)]bisbenzene | 0.46 | 0.24 |
| Area under curve | Tetracosahexaenoic acid | 0.32 | 0.31 |
| Area under curve | (3R, 6'Z)-3,4-dihydro-8-hydroxy-3-(6-pentadecenyl)-1H-2-benzopyran-1-one | 0.56 | 0.19 |
| Area under curve | Cervonoyl ethanolamide | 0.29 | 0.34 |
| Area under curve | 12a-hydroxy-3-oxocholadienic acid | 0.29 | 0.34 |
| Area under curve | 7-hydroxy-3-oxocholanoic acid | 0.42 | 0.26 |
| Area under curve | Acetoxy-[10]-gingerol | 0.56 | 0.19 |
| Area under curve | Piritramide | 0.51 | 0.21 |
| Area under curve | 2-(2-methylbutanoyl)-9-(3-methyl-2E-pentenoyl)-2b,9a-dihydroxy-4Z,10(14)-oplopadien-3-one | 0.53 | 0.20 |
| Area under curve | Erdafitinib | 0.16 | 0.43 |
| Area under curve | Cholesteryl chloride | 0.73 | 0.11 |
| Area under curve | Cholylserine | 0.76 | 0.10 |
| Area under curve | Deoxycholylmethionine | 0.81 | 0.08 |
| Area under curve | Ilmofosine | 0.37 | 0.29 |
| Area under curve | Deoxycholylphenylalanine | 0.80 | 0.08 |
| Area under curve | Chenodeoxycholylphenylalanine | 0.46 | 0.24 |
| Area under curve | SM(d18:2(4E,14Z)/20:4(8Z,11Z,14Z,17Z)-2OH(5S,6R)) | 0.50 | 0.22 |
| Area under curve | SM(d18:1/PGJ2) | 0.53 | 0.20 |
| Area under curve | SM(d18:2(4E,14Z)/PGD1) | 0.42 | 0.26 |
| Area under curve | SM(d18:2(4E,14Z)/PGE1) | 0.39 | 0.27 |
| Area under curve | SM(d18:1/TXB2) | 0.35 | 0.29 |
| Area under curve | PG(22:5(4Z,7Z,10Z,13Z,19Z)-O(16,17)/i-17:0) | 0.42 | 0.26 |
| Area under curve | PE-NMe(22:6(4Z,7Z,10Z,13Z,16Z,19Z)/20:3(5Z,8Z,11Z)) | 0.65 | 0.15 |
| Area under curve | PC(16:1(9Z)/22:6(5Z,8E,10Z,13Z,15E,19Z)-2OH(7S, 17S)) | 0.73 | 0.11 |
| Area under curve | PG(22:5(4Z,7Z,10Z,13Z,19Z)-O(16,17)/i-18:0) | 0.43 | 0.25 |
| Area under curve | PG(22:6(4Z,7Z,10Z,13E,15E,19Z)-OH(17)/i-18:0) | 0.18 | 0.41 |
| Area under curve | PG(20:4(5Z,8Z,11Z,14Z)-OH(19S)/20:1(11Z)) | 0.30 | 0.33 |
| Area under curve | PS(PGF1ɑ/22:5(7Z,10Z,13Z,16Z,19Z)) | 0.51 | 0.21 |
| Area under curve | PGP(18:1(9Z)-O(12,13)/22:5(7Z,10Z,13Z,16Z,19Z)) | 0.50 | 0.22 |
| Blood glucose | 1-phenyl-1-cyclohexene | 0.03 | 0.61 |
| Blood glucose | Fadrozole | 0.03 | 0.64 |
| Blood glucose | 15-octadecene-9,11,13-triynoic acid | 0.11 | 0.65 |
| Blood glucose | 1,1'-[1,12-dodecanediylbis(oxy)]bisbenzene | 0.09 | 0.48 |
| Blood glucose | Tetracosahexaenoic acid | 0.02 | 0.51 |
| Blood glucose | (3R, 6'Z)-3,4-dihydro-8-hydroxy-3-(6-pentadecenyl)-1H-2-benzopyran-1-one | 0.05 | 0.58 |
| Blood glucose | Cervonoyl ethanolamide | 0.13 | 0.62 |
| Blood glucose | 12a-hydroxy-3-oxocholadienic acid | 0.05 | 0.75 |
| Blood glucose | 7-hydroxy-3-oxocholanoic acid | 0.03 | 0.47 |
| Blood glucose | Acetoxy-[10]-gingerol | <0.01 | 0.57 |
| Blood glucose | Piritramide | 0.04 | 0.60 |
| Blood glucose | 2-(2-methylbutanoyl)-9-(3-methyl-2E-pentenoyl)-2b,9a-dihydroxy-4Z,10(14)-oplopadien-3-one | 0.17 | 0.71 |
| Blood glucose | Erdafitinib | 0.07 | 0.67 |
| Blood glucose | Cholesteryl chloride | <0.01 | 0.63 |
| Blood glucose | Cholylserine | 0.02 | 0.42 |
| Blood glucose | Deoxycholylmethionine | 0.03 | 0.54 |
| Blood glucose | Ilmofosine | 0.06 | 0.60 |
| Blood glucose | Deoxycholylphenylalanine | 0.04 | 0.64 |
| Blood glucose | Chenodeoxycholylphenylalanine | 0.05 | 0.62 |
| Blood glucose | SM(d18:2(4E,14Z)/20:4(8Z,11Z,14Z,17Z)-2OH(5S,6R)) | 0.03 | 0.68 |
| Blood glucose | SM(d18:1/PGJ2) | 0.03 | 0.56 |
| Blood glucose | SM(d18:2(4E,14Z)/PGD1) | <0.01 | 0.59 |
| Blood glucose | SM(d18:2(4E,14Z)/PGE1) | 0.04 | 0.57 |
| Blood glucose | SM(d18:1/TXB2) | 0.06 | 0.78 |
| Blood glucose | PG(22:5(4Z,7Z,10Z,13Z,19Z)-O(16,17)/i-17:0) | <0.01 | 0.62 |
| Blood glucose | PE-NMe(22:6(4Z,7Z,10Z,13Z,16Z,19Z)/20:3(5Z,8Z,11Z)) | 0.05 | 0.56 |
| Blood glucose | PC(16:1(9Z)/22:6(5Z,8E,10Z,13Z,15E,19Z)-2OH(7S, 17S)) | 0.09 | 0.57 |
| Blood glucose | PG(22:5(4Z,7Z,10Z,13Z,19Z)-O(16,17)/i-18:0) | 0.03 | 0.51 |
| Blood glucose | PG(22:6(4Z,7Z,10Z,13E,15E,19Z)-OH(17)/i-18:0) | 0.09 | 0.51 |
| Blood glucose | PG(20:4(5Z,8Z,11Z,14Z)-OH(19S)/20:1(11Z)) | 0.06 | 0.56 |
| Blood glucose | PS(PGF1ɑ/22:5(7Z,10Z,13Z,16Z,19Z)) | 0.06 | 0.56 |
| Blood glucose | PGP(18:1(9Z)-O(12,13)/22:5(7Z,10Z,13Z,16Z,19Z)) | 0.05 | 0.57 |
| CHO | 1-phenyl-1-cyclohexene | 0.03 | 0.34 |
| CHO | Fadrozole | 0.28 | 0.29 |
| CHO | 15-octadecene-9,11,13-triynoic acid | 0.35 | 0.54 |
| CHO | 1,1'-[1,12-dodecanediylbis(oxy)]bisbenzene | 0.07 | 0.37 |
| CHO | Tetracosahexaenoic acid | 0.24 | 0.45 |
| CHO | (3R, 6'Z)-3,4-dihydro-8-hydroxy-3-(6-pentadecenyl)-1H-2-benzopyran-1-one | 0.03 | 0.48 |
| CHO | Cervonoyl ethanolamide | 0.14 | 0.57 |
| CHO | 12a-hydroxy-3-oxocholadienic acid | 0.11 | 0.20 |
| CHO | 7-hydroxy-3-oxocholanoic acid | 0.05 | 0.55 |
| CHO | Acetoxy-[10]-gingerol | 0.54 | 0.46 |
| CHO | Piritramide | 0.05 | 0.29 |
| CHO | 2-(2-methylbutanoyl)-9-(3-methyl-2E-pentenoyl)-2b,9a-dihydroxy-4Z,10(14)-oplopadien-3-one | 0.06 | 0.23 |
| CHO | Erdafitinib | 0.02 | 0.46 |
| CHO | Cholesteryl chloride | 0.13 | 0.15 |
| CHO | Cholylserine | 0.35 | 0.41 |
| CHO | Deoxycholylmethionine | 0.47 | 0.55 |
| CHO | Ilmofosine | 0.13 | 0.53 |
| CHO | Deoxycholylphenylalanine | 0.63 | 0.62 |
| CHO | Chenodeoxycholylphenylalanine | 0.19 | 0.46 |
| CHO | SM(d18:2(4E,14Z)/20:4(8Z,11Z,14Z,17Z)-2OH(5S,6R)) | 0.06 | 0.62 |
| CHO | SM(d18:1/PGJ2) | 0.08 | 0.50 |
| CHO | SM(d18:2(4E,14Z)/PGD1) | 0.13 | 0.58 |
| CHO | SM(d18:2(4E,14Z)/PGE1) | 0.10 | 0.68 |
| CHO | SM(d18:1/TXB2) | 0.04 | 0.21 |
| CHO | PG(22:5(4Z,7Z,10Z,13Z,19Z)-O(16,17)/i-17:0) | 0.51 | 0.34 |
| CHO | PE-NMe(22:6(4Z,7Z,10Z,13Z,16Z,19Z)/20:3(5Z,8Z,11Z)) | 0.28 | 0.49 |
| CHO | PC(16:1(9Z)/22:6(5Z,8E,10Z,13Z,15E,19Z)-2OH(7S, 17S)) | 0.11 | 0.61 |
| CHO | PG(22:5(4Z,7Z,10Z,13Z,19Z)-O(16,17)/i-18:0) | 0.09 | 0.51 |
| CHO | PG(22:6(4Z,7Z,10Z,13E,15E,19Z)-OH(17)/i-18:0) | <0.01 | 0.70 |
| CHO | PG(20:4(5Z,8Z,11Z,14Z)-OH(19S)/20:1(11Z)) | 0.24 | 0.37 |
| CHO | PS(PGF1ɑ/22:5(7Z,10Z,13Z,16Z,19Z)) | 0.15 | 0.44 |
| CHO | PGP(18:1(9Z)-O(12,13)/22:5(7Z,10Z,13Z,16Z,19Z)) | 0.33 | 0.31 |

CHO: cholesterol.

# Table A.15 Spearman rank correlation results between the blood glucose/lipid parameters and the differential serum metabolites.

| **Blood glucose/lipid parameter** | **Differential serum metabolites** | **P** | **r** |
| --- | --- | --- | --- |
| Area under curve | Benzeneacetic acid, 4-(2-(diethylamino)-2-oxoethoxy)-3-ethoxy-, propyl ester | 0.97 | -0.01 |
| Area under curve | LysoPE(24:0/0:0) | 0.78 | -0.09 |
| Area under curve | N-acetyl-L-phenylalanyl-3,5-diiodo-L-tyrosine | 0.39 | -0.27 |
| Area under curve | PA(18:3(9,11,15)-OH(13)/i-18:0) | 0.29 | -0.34 |
| Area under curve | Sagerinic acid | 0.42 | -0.26 |
| Blood glucose | Benzeneacetic acid, 4-(2-(diethylamino)-2-oxoethoxy)-3-ethoxy-, propyl ester | 0.08 | -0.52 |
| Blood glucose | LysoPE(24:0/0:0) | 0.02 | -0.64 |
| Blood glucose | N-acetyl-L-phenylalanyl-3,5-diiodo-L-tyrosine | 0.04 | -0.61 |
| Blood glucose | PA(18:3(9,11,15)-OH(13)/i-18:0) | 0.07 | -0.54 |
| Blood glucose | Sagerinic acid | <0.01 | -0.80 |
| CHO | Benzeneacetic acid, 4-(2-(diethylamino)-2-oxoethoxy)-3-ethoxy-, propyl ester | 0.10 | -0.50 |
| CHO | LysoPE(24:0/0:0) | 0.12 | -0.48 |
| CHO | N-acetyl-L-phenylalanyl-3,5-diiodo-L-tyrosine | 0.25 | -0.36 |
| CHO | PA(18:3(9,11,15)-OH(13)/i-18:0) | 0.05 | -0.57 |
| CHO | Sagerinic acid | 0.31 | -0.32 |

CHO: cholesterol.
